# Peripheral monocytes and neutrophils promote photoreceptor cell death in an experimental retinal detachment model

**DOI:** 10.1038/s41419-023-06350-6

**Published:** 2023-12-16

**Authors:** Daniel E. Maidana, Lucia Gonzalez-Buendia, Sara Pastor-Puente, Afsar Naqvi, Eleftherios Paschalis, Andrius Kazlauskas, Joan W. Miller, Demetrios G. Vavvas

**Affiliations:** 1 Retina Service, Angiogenesis Lab, Massachusetts Eye and Ear Infirmary, Harvard Medical School, Boston, MA USA; 2 Department of Ophthalmology and Visual Sciences, Illinois Eye and Ear Infirmary, University of Illinois at Chicago, Chicago, IL USA; 3 Department of Physiology and Biophysics, University of Illinois at Chicago, Chicago, IL USA; 4 Mucosal Immunology Lab, College of Dentistry, University of Illinois at Chicago, Chicago, IL USA

**Keywords:** Cell death, Visual system

## Abstract

Photoreceptor cell death and immune cell infiltration are two major events that contribute to retinal degeneration. However, the relationship between these two events has not been well delineated, primarily because of an inadequate understanding of the immunological processes involved in photoreceptor degeneration, especially that of peripheral leukocytes that infiltrate the subretinal space and retinal tissues. In this work, we characterized the role of leukocyte infiltration within the detached retina. We observed that CD45^+^ CD11b^+^ Ly6G^+^ neutrophils and CD45^+^ CD11b^+^ Ly6G^−^ Ly6C^+^ monocytes are the predominant peripheral immune cell populations that infiltrate the retinal and subretinal space after detachment. Selective depletion of monocytes or neutrophils using cell-specific targeting is neuroprotective for photoreceptors. These results indicate that peripheral innate immune cells contribute to photoreceptor degeneration, and targeting these immune cell populations could be therapeutic during retinal detachment.

## Introduction

The detachment of the neurosensory retina from the retinal pigment epithelium/choriocapillaris (RPE/Ch), can occur as a primary entity or complicate the most prevalent retinal diseases, including age-related macular degeneration (AMD), proliferative diabetic retinopathy (PDR), or retinopathy of prematurity (ROP). Besides the occurrence of retinal detachment (RD) in these diverse conditions, subretinal delivery of photoreceptor/RPE gene therapy is a therapeutic strategy that induces a localized detachment of the retina [1, 2]. Despite the different etiologies of RD, the separation of the neurosensory retina from its oxygen and nutrient supply compromises photoreceptor viability [3–6]. In parallel, a cellular and cytokine/chemokine inflammatory immune response occurs in the detached retina [7, 8]. Moreover, the ongoing photoreceptor cell death and inflammation build-up from the initial injury can further compromise visual function.

The relationship between cell death and inflammation is intricately entwined and complex. Whether the crosstalk of these biological pathways contributes to disease pathology has not yet been well delineated, mostly because their corresponding roles have been addressed unidirectionally; either from the point of view of photoreceptor cell death causing inflammation or from inflammation compromising photoreceptor viability. Specifically, certain forms of cell death pathways operative in retinal detachment, such as necroptosis, can induce inflammation and activation of cellular immune mechanisms [7–13]. In turn, inflammation can create a detrimental milieu by generating reactive oxygen species, enzymes, and cytokines, which can further compromise cell viability [7, 14]. This bidirectional relationship has been a significant obstacle in our understanding of photoreceptor cell death [14]. During retinal detachment, peripheral leukocytes infiltrate the subretinal space and retinal tissues [8, 15]. However, it remains unknown whether such infiltration of the retina can be detrimental or protective for photoreceptors in retinal detachment.

In this work, we aimed to study the role of innate immunity in modulating photoreceptor cell death. Using a murine experimental RD model and bone marrow transplantation, we characterized the cellular infiltration of peripheral myeloid cells in the retina. Selective depletion of peripheral monocyte and neutrophil populations demonstrated that these cells are detrimental to photoreceptor survival in retinal detachment. These findings will advance our understanding of the multifactorial effects that lead to photoreceptor cell death and vision impairment.

## Materials and methods

All reagents used in this work are listed in Supplementary Table 1 with their respective manufacturer and catalog number.

### Animals and breeding

All animals used in experiments and breeding adhered to the statement of the Association for Research in Vision and Ophthalmology (ARVO). Animal protocols were reviewed and approved by the Animal Care Committee of the Massachusetts Eye and Ear Infirmary and the University of Illinois Chicago. C57BL/6J, CCR2^RFP/+^ CX3CR1^GFP/+^, and C57BL/6-Tg(CAG-EGFP)131Osb/LeySopJ (EGFP) were purchased from The Jackson Laboratories. Eight-week-old male and female mice were used for experiments. Animals were randomly allocated to control and experimental groups. Mice were maintained in a standard 12-h light/dark cycle and fed a supply of standard chow ad libitum.

### Bone marrow isolation, culture, and transplantation

Three- to four-week-old recipient mice were conditioned with intraperitoneal busulfan injections as previously described [16, 17]. In brief, animals received myelo-conditioning therapy with 105 mg/kg of busulfan before bone marrow transplantation (BMT) of hematopoietic precursors. Bone marrow progenitors were injected via the tail vein (2 × 10^7^cells). Four weeks after BMT, we assessed immune reconstitution in the peripheral blood samples of chimeric mice by flow cytometry. Bone marrow chimeras with ≥95% immune reconstitution were used for experiments. For in vitro experiments, bone marrow-derived macrophages were cultured as previously described [18].

### Experimental retinal detachment model

Eight-week-old mice were anesthetized with a mixture of 2,2,2-tribromoethanol and 2- methyl-2-butanol at a dose of 125 mg/kg or a mixture of ketamine (80 mg/kg) and xylazine (5 mg/kg) via intraperitoneal injection. Retinal detachment was induced via subretinal injection with 1% sodium hyaluronate, as previously described [19]. Eyes with successful RD at endpoint were included. Eyes with hemorrhage, leakage, or cataracts were excluded from further analysis. The investigator (DEM) was masked to the group allocation when performing the subretinal injection.

### Immunohistochemistry and imaging

Eyes were enucleated and embedded in Tissue-Tek Optimal Cutting Temperature compound. Eyecups were sectioned to 10 µm axial cryosections starting at 800 µm from the eye and stored at −80 °C until used. Primary antibodies were incubated overnight at 4 °C. Secondary were incubated for 2 h at room temperature. Slides were counterstained with DAPI and mounted with Fluoromount-G mounting medium. Terminal deoxynucleotidyl transferase (dUTP) nick end labeling (TUNEL) assay was performed with ApopTag fluorescein direct in situ apoptosis detection kit according to the manufacturer’s instructions. TUNEL^+^ cells were counted automatedly in ImageJ (version 1.52, http://imagej.nih.gov/ij/; provided in the public domain by the National Institutes of Health, Bethesda, MD) using the TUNEL Cell Counter plugin [20]. 4′,6-diamidino-2-phenylindole (DAPI) was used as a nuclear counterstaining. Antibodies were validated a priori by immunofluorescence in spleens or lymph nodes. Sections were imaged with a ZEISS Axio (Thornwood, NY) epifluorescence microscope or ZEISS LSM 800 laser confocal microscope.

### Flow cytometry analyses

Single-cell suspensions from the detached retina were obtained using the Papain Dissociation System (Worthington, Columbus, OH), as previously described. Live/dead exclusion assay (Zombie NIR, BioLegend, San Diego, CA) and DAPI staining were used to identify nucleated live singlets. CD11b, CD45, Ly6G, Ly6C antibodies were purchased from BioLegend (CD11b clone M1/70, #101243), and BD Biosciences (CD45 clone 30-F11, #564279; (Ly6G clone 1A8, #560601; and Ly6C clone HK1.4, #755194). Fluorescence-minus-one and single stain controls were acquired for each antibody or fluorescent reporter. Samples were analyzed with an Aurora Spectral Cytometer (Cytek Biosciences, Fremont, CA). Data analysis was performed with Kaluza cytometry software (Beckman Coulter, Brea, CA).

### Peripheral monocyte/macrophage and neutrophil depletion

For peripheral monocyte/macrophage depletion, animals received an intraperitoneal injection of 200 µL clodronate liposomes or saline 4 and 2 days prior and on the day of retinal detachment. A volume of 100 µL clodronate liposomes or saline was injected 2-, 4- and 6 days following RD. CD11b^+^ cell depletion was assessed in the subretinal space. For peripheral neutrophil depletion, animals received an intraperitoneal injection of 400 µg InVivoPlus anti-mouse Ly6G (Clone 1A8) antibody (BioxCell, #BP0075-1) or saline 2 days before retinal detachment, on the day of RD, and 2, 4 and 6 days following RD. Ly6G^+^ cell depletion was assessed in the subretinal space of the detached retinas.

### Statistical analyses

Statistical analyses were performed with SAS Software (2016, SAS Institute Inc., Cary, NC). Normality was assessed with the Shapiro–Wilk test. Equal variances were assumed given group sizes. Sample size for photoreceptor cell count was chosen based on prior work [21]. Statistical significance for differences between groups was determined with a two-sided *T*-test or Student’s *T*-test for two-group comparisons or one-way ANOVA with Tukey post-hoc correction for multiple comparisons. Results are expressed as mean ± standard error of the mean (SEM). A *p*-value of ≤0.05 was considered statistically significant.

## Results

### Retinal detachment induces early and sustained CX3CR1+ and CCR2+ infiltration of the retina

**Fig. 1:**
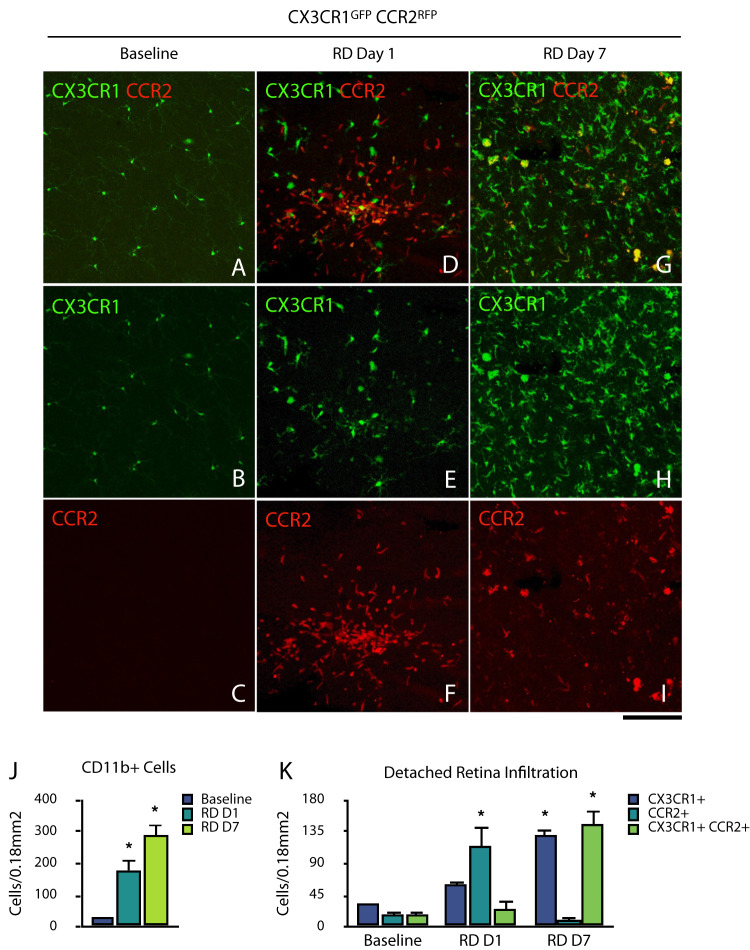
CX3CR1+ and CCR2+ infiltration in retinal detachment. **A**–**I** Recruitment of CCR2/CX3CR1-expressing cells in detached retina wholemounts. Images are representative of ≥3 experiments. Scale bar 50 µm (**A**–**I**). **J** Quantitation of CD11b^++^ cells in the detached retina (*n* = 2, 3, 3). **K** Quantitation of CD11b^+^, CX3CR1^+^, and CCR2^+^ cells in the detached retina (*n* = 2, 3, 3). *p < 0.05.

Detachment of the neurosensory retina induces significant neuroinflammation [8, 10, 11, 22]. However, the longitudinal local and peripheral cellular infiltration are poorly understood. For this purpose, we performed retinal detachments in CX3CR1^GFP+^ CCR2^RFP+^ transgenic mice to explore the longitudinal recruitment of CD11b^+^ CX3CR1 and CCR2-expressing cells in the detached retina (Fig. 1A–I). We observed that in a steady state, CX3CR1^GFP +^ retinal microglia have marginal CCR2 expression, as previously described [23]. Following RD, there was a significant >6-fold increase in the total CD11b^+^ retinal cellularity on day 1 (*p* = 0.011), which increased to >9-fold on day 7 (*p* = 0.001), with ubiquitous cellular infiltration and few acellular lacunae, which suggest continuous infiltration from peripheral leukocytes (Fig. 1J). On day 1, we observed a predominant infiltrating population of CD11b^+^ CCR2^RFP+^ cells (116.20 ± 58.59 cells, *p* = 0.006), followed by CD11b^+^ CC3CR1^GFP+^ (60.60 ± 21.06 cells, *p* = 0.241) and a small subset of CD11b^+^ CC3CR1^GFP+^ CCR2^RFP+^ cells (26.60 ± 23.39 cells, *p* = 0.777) (Fig. 1A–I, K). On day 7 after RD, the predominant population were CD11b^+^ CC3CR1^GFP+^ CCR2^RFP+^ (147.40 ± 36.80 cells, *p* < 0.001), followed by CD11b^+^ CC3CR1^GFP+^ (129.40 ± 35.78 cells, *p* = 0.002), and ultimately a scarce population of CD11b^+^ CCR2^RFP+^ (9.40 ± 9.20 cells, *p* = 0.0.782) (Fig. 1A–I, K). Collectively, these results indicate that the early phase of RD is characterized by an overwhelming majority of infiltrating CCR2^+^ inflammatory cells, likely from the peripheral compartment. On day 7, these infiltrating cells continue to show an inflammatory CX3CR1^+^ CCR2^+^ signature.

### Peripheral leukocytes progressively infiltrate the detached retina

**Fig. 2:**
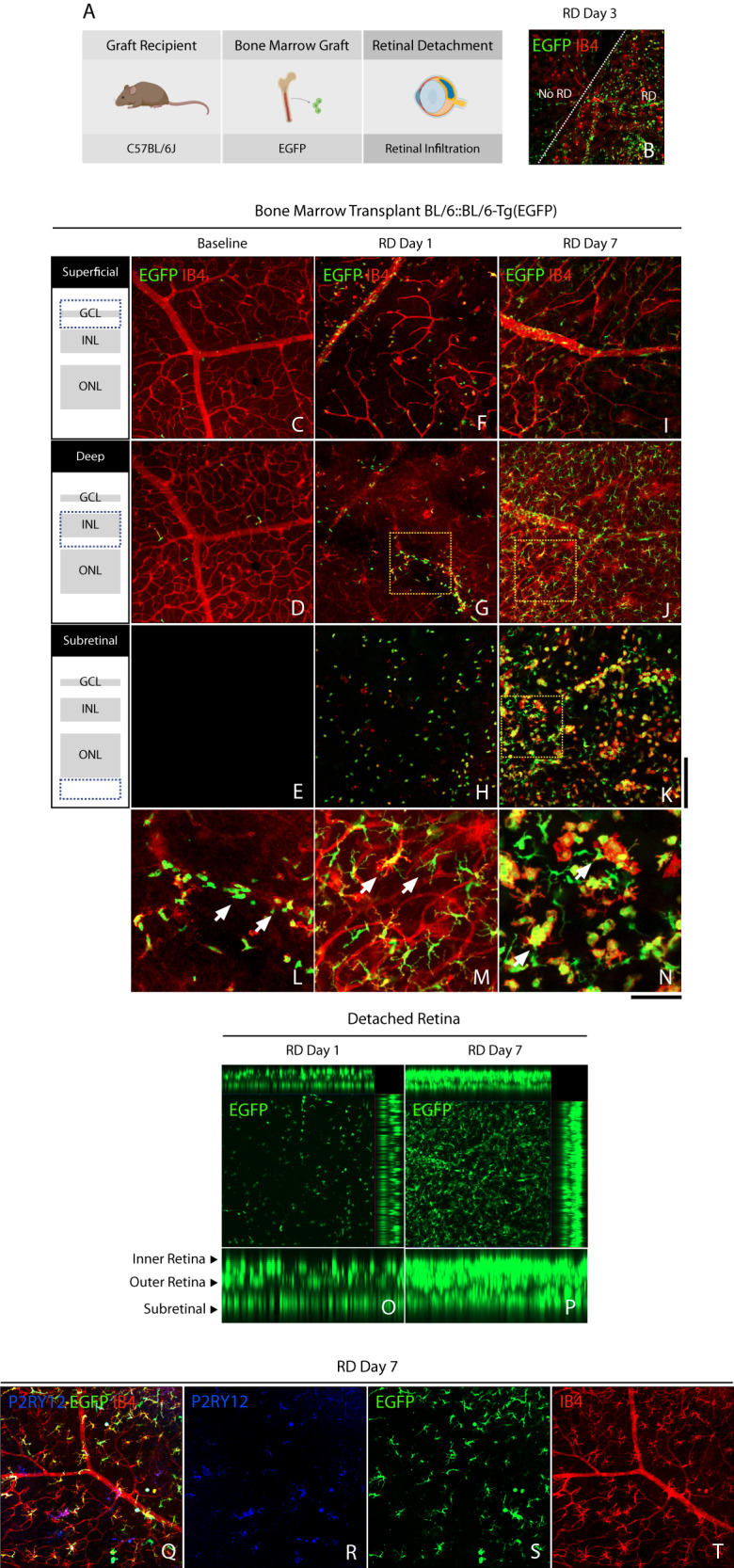
Peripheral myeloid cell infiltration in retinal detachment. **A** Outline of recipients, bone marrow transplantation, and experimental retinal detachment model (Created with BioRender.com). **B** Representative image of retinal wholemounts of chimeric mice at day 3 following retinal detachment showing the attached (No RD) and detached retina (RD) separated by a white dotted line. **C**–**E** Representative confocal image of chimeric mice at baseline. **F**–**H** Detached retina at day 1, with moderate peripheral EGFP^+^ BM-derived infiltration of the retina and subretinal space. **I**–**K** Detached retina at day 7, with significant EGFP^+^ infiltration of the retina. **L** Magnification of image **G** (dotted box), showing early retinal infiltration by peripheral EGFP^+^ cells of ameboid shape (arrows). **M** Magnification of image (**J**) (dotted box), showing substantial retinal infiltration by EGFP^+^ cells with ramified phenotype (arrows). **N** Magnification of image (**K**) (dotted box), showing mixed ameboid and ramified morphology on EGFP^+^ cells in the subretinal space. **O** and **P** Z-projection of confocal images showing peripheral EGFP^+^ cell infiltration in the detached retina and subretinal space on days 1 and 7. **Q**–**T** Scarce P2RY12^+^ retinal microglia surrounded by ubiquitous myeloid-derived EGFP+ cells in the detached retina. Images are representative of ≥3 experiments. Scale bar 100 µm (**A**–**I**).

Next, we sought to elucidate the peripheral leukocyte contribution to the increased cellularity in the detached retina. Given technical limitations to identifying retinal microglia from peripheral monocytes/macrophages, and that CCR2 and CX3CR1 expression cannot separate these cells upon injury, we generated chimeric mice with bone marrow transplantation (BMT) of EGFP^+^ hematopoietic precursors (Fig. 2). Following successful immune reconstitution, we observed a remarkable EGFP^+^ myeloid-derived infiltration after RD (Fig. 2B). Laser confocal Z-plane projections in naïve eyes confirmed the absence of peripheral infiltration of the retina in naïve animals, which only showed EGFP^+^ cells at the vascular lumen likely representing patrolling cells (Fig. 2C–E). One day after RD, detached retinas showed incipient infiltration of EGFP^+^ cells in the superficial, deep, and subretinal space layers, with a predominant ameboid phenotype within retinal layers (Fig. 2F–H, L). Seven days following RD, detached retinas showed consistent infiltration of EGFP^+^ cells at the superficial layer, but mostly at deep retinal layers and subretinal space (Fig. 2I–K, M and N). In contrast to day 1, infiltrated EGFP^+^ cells displayed a ramified morphology at the retinal layers while a combination of ameboid and ramified in the subretinal space. Orthogonal projection of the detached retina showed an overwhelming majority of EGFP+ cells in all layers on day 7, compared to day 1 after RD (Fig. 2O and P). Interestingly, at day 7, these predominant myeloid-derived EGFP^+^ cells interacted with scarce P2RY12-expressing microglia (Fig. 2Q–T). These results indicate that peripheral myeloid cells are a major contributor to the progressive infiltration of the superficial, deep, and subretinal layers following retinal detachment. Moreover, these results suggest that myeloid cells infiltrate and display different morphology as a function of time and location in the retina. Taken together, these results suggest that peripheral myeloid cells are the predominant population in late RD and can potentially play important roles by interacting with tissue-resident macrophages/microglia.

### Ly6G^+^ and Ly6C^+^ cells infiltrate the retina early after retinal detachment

**Fig. 3:**
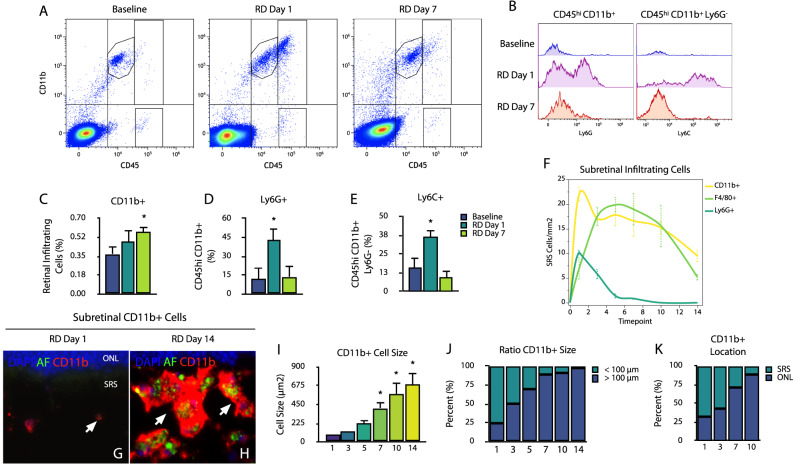
Immunophenotyping of infiltrating cells in retinal detachment. **A** Density plots and gating strategy from single-cell suspensions of live CD45 CD11b-infiltrating cells at baseline, days 1 and day 7 after retinal detachment. **B** Histogram overlays of CD45^hi^ CD11b^+^ Ly6G^+^ and CD45^hi^ CD11b^+^ Ly6G^−^ Ly6C^+^ cells. **C**–**E** Quantitative analyses of total CD11b^+^, CD45^hi^ CD11b^+^ Ly6G^+^, and CD45^hi^ CD11b^+^ Ly6G^−^ Ly6C^+^(pooled samples, *n* = 6 per replicate). **F** Quantitation of subretinal cells in the subretinal space of whole-eye cryosection images (*n* = 4 per group). Images are representative of ≥2 experiments. **G** and **H** Representative retinal cryosection image of subretinal CD11b^+^ cells at day 1 and 14, showing cell size increase (*n* = 4, 4). **I** Subretinal CD11b^+^ cell size ratio thorough days 1–14 after detachment. **J** Ratio of CD11b^+^ >100 µm^2^ area. **K** Location of subretinal CD11b^+^ in the subretinal space (SRS) or in contact with the outer nuclear layer (ONL) (*n* = 4 per group). *p < 0.05.

Given the observed leukocyte recruitment in the detached retina, we sought to further characterize the myeloid infiltrating cells, which can include neutrophils (Ly6G), monocytes/macrophages (Ly6C, F4/80), and dendritic cells (CD11c). For this purpose, we investigated the immunophenotypes in the detached retina by spectral flow cytometry (Fig. 3A, B). We found that the total CD11b+ fold-change was less pronounced compared to that observed with retinal wholemounts, likely due to sample processing (day 7, *p* = 0.28) (Fig. 3C). We observed that CD45^+^ CD11b^+^ Ly6G^+^ cells significantly increased (3.5-fold) at day 1 (*p* = 0.005) and returned to similar baseline levels by day 7 (Fig. 3D). Similarly, CD45^+^ CD11b^+^ Ly6G^−^ Ly6C^+^ cells significantly increased ~2-fold at day 1 (*p* = 0.002) and decreased beyond baseline on day 7 (*p* < 0.001 vs. day 1) (Fig. 3E). These results suggest that CD45^+^ CD11b^+^ cells at day 1 after RD likely represent Ly6G^+^ polymorphonuclear neutrophils and Ly6C^+^ infiltrating monocytes/macrophages.

To further confirm these findings, we investigated the subretinal space infiltration in whole-eye cryosection immunofluorescence (Fig. 3A), since cells suspended in sodium hyaluronate may be lost during sample isolation/processing for flow cytometry. We observed that CD11b^+^, F4/80^+^, and Ly6G^+^ cells infiltrated this space from day 1 to day 14 following RD (Fig. 3F). We did not observe CD3^+^ T-lymphocytes, B220^+^ B-lymphocytes, NCR1^+^ natural killer cells, or CD11c^+^ dendritic cells in the subretinal space. Among the subretinal space infiltrating cells, Ly6G^+^ cells were significantly increased on day 1 (*p* < 0.001) and day 3 (*p* < 0.001), and declined during the first week, and were absent by day 14. In contrast, F4/80^+^ cells peaked at day 3 (*p* = 0.007), and were consistently detected at day 5 (*p* = 0.007), day 7 (*p* = 0.004), and day 10 (*p* = 0.014). As expected, the total CD11b^+^ cell count parallels the cumulative Ly6G^+^ and F4/80^+^ cell count. Interestingly, the surface area of subretinal CD11b^+^ cells significantly increased from 83.11 µm^2^ on day 1 to 407.50 µm^2^ on day 7 (*p* = 0.021), 571.52 µm^2^ on day 10 (*p* < 0.001), and 683.00 µm^2^ on day 14 (*p* < 0.001) (Fig. 3G–I). Moreover, the number of CD11b^+^ cells >100 µm represented a minority of the subretinal pool on day 1 (24%) and shifted to a vast majority of CD11b^+^ cells >100 µm on day 14 (98%) (Fig. 3J). In addition to the size increase, on day 1 following RD, the largest percent of CD11b^+^ cells were located freely in the subretinal space (67%) (Fig. 3K). This proportion reversed by day 7 and by day 10, 88% of all subretinal CD11b^+^ cells were in contact with the retina. Taken together, these results suggest that subretinal CD11b^+^, Ly6G^+^, and F4/80^+^ cells are the primary infiltrating populations, most likely representing peripheral neutrophils and monocytes/macrophages. In addition, CD11b^+^ cells increase their size and localize close to photoreceptors as the detachment persists, which may reflect continuous phagocytosis of cellular debris and photoreceptor segments. In turn, this phenotype may contribute to their persistence in the subretinal space.

### Peripheral monocyte/macrophage depletion protects photoreceptors after retinal detachment

**Fig. 4:**
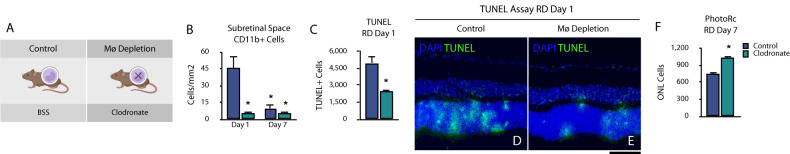
Peripheral monocyte/macrophage depletion protects photoreceptors after retinal detachment. **A** Outline of recipients, peripheral monocyte/macrophage depletion, and experimental retinal detachment model (Created with BioRender.com). **B** Subretinal infiltration of CD11b^+^ cells in control and clodronate groups after retinal detachment in retinal cryosections. **C** TUNEL assay in the detached retina in control and clodronate groups at day 1 after retinal detachment. **D** and **E** Representative cryosection image of TUNEL assay in control and clodronate groups at day 1 after retinal detachment. **F** Photoreceptor cell count in the detached retina in control and clodronate groups at day 7. Images are representative of ≥ 3 experiments (*n* = 4, 10). *p < 0.05. Scale bar 50 µm (**D** and **E**).

We sought to elucidate the role of infiltrating monocytes/macrophages in photoreceptor cell death. Given the peak of cell death in this model on day 1 [19, 21] and the observed early CD45^+^ CD11b^+^ Ly6G^−^ Ly6C^+^ infiltration at this timepoint, we hypothesized that monocyte/macrophages infiltration may contribute to early photoreceptor cell death. For this purpose, we depleted peripheral monocytes/macrophages with clodronate liposomes (Fig. 4A) before RD. Of note, systemic clodronate liposomes do not cross the blood barrier and do not deplete retinal microglia [24–26]. Compared to baseline, monocyte/macrophage depletion in mice showed a significant reduction of infiltrating CD11b^+^ at day 1 (87%, *p* < 0.001) and day 7 following RD compared to baseline (Fig. 4B). We observed a significant reduction of TUNEL^+^ ONL cells on day 1 after RD in monocyte/macrophage-depleted mice (*p* = 0.011) (Fig. 4C–E). Moreover, monocyte/macrophage-depleted mice showed increased photoreceptor survival on day 7 after RD (1032.30 ± 126.66 vs. 747.40 ± 75.80, *p* < 0.001) ((Fig. 4F). Collectively, these results indicate that peripheral monocyte/macrophage depletion in mice significantly reduces subretinal space CD11b^+^ infiltration and is neuroprotective to photoreceptors in this experimental retinal detachment model.

### Peripheral neutrophil depletion protects photoreceptors after retinal detachment

**Fig. 5:**
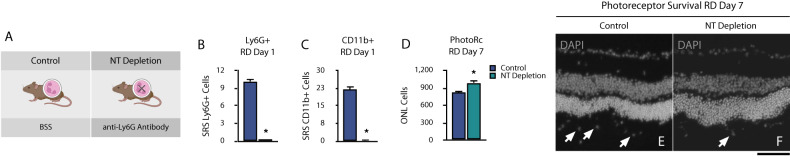
Peripheral neutrophil depletion protects photoreceptors after retinal detachment. **A** Outline of recipients, peripheral neutrophil depletion, and experimental retinal detachment model (Created with BioRender.com). **B** and **C** Subretinal infiltration of CD11b^**+**^ and Ly6G^**+**^ cells in control and anti-Ly6G^+^ neutralizing antibody groups after retinal detachment in retinal cryosections. **D** Photoreceptor cell count in the detached retina in control and anti-Ly6G^**+**^ groups at day 7. **E** and **F** Representative cryosection image of outer nuclear layer and subretinal cells (arrows) in both groups at day 7 after retinal detachment. Images are representative of ≥ 3 experiments (*n* = 5, 7). *p < 0.05. Scale bar 50 µm (**E** and **F**).

Considering that CD45^+^ CD11b^+^ Ly6G^+^ cells similarly infiltrate the retinal and subretinal space early on retinal detachment, we investigated their contribution to photoreceptor cell death. We systemically depleted peripheral Ly6G^+^ cells with a monoclonal murine anti-Ly6G antibody before RD (Fig. 5A). We used an antibody clone (1A8) which can selectively reduce peripheral blood neutrophils but not monocytes [27]. Preemptive and post-RD treatment with anti-Ly6G neutralizing antibody showed a significant depletion of subretinal Ly6G^+^ cells (97.93%, *p* < 0.001) at day 1 following RD (Fig. 5B). CD11b^+^ cells showed a similar reduction at this timepoint (99.04%, *p* = 0.003) (Fig. 5C). We observed that Ly6G^+^ neutrophil depletion increased photoreceptor survival on day 7 RD (989.00 ± 1123.10 vs. 813.00 ± 110.99, *p* < 0.028) (Fig. 5D–F). Taken together, these results indicate that peripheral Ly6G^+^ cell depletion significantly reduces the overall subretinal space cellular infiltration and is neuroprotective for photoreceptors in the detached retina.

## Discussion

In this work, we demonstrated that peripheral leukocyte infiltration in the detached retina is a significant contributor to photoreceptor cell death. This immune cell infiltration is ubiquitous in the detached retina, affecting superficial, deep, and subretinal layers. Moreover, the selective depletion of monocytes/macrophages or neutrophils reduced the infiltrating cells in the detached subretinal space. Finally, this work demonstrates that modulation of these cell populations is neuroprotective for photoreceptors in the detached retina.

The detachment of the retina induces a significant chemoattractant stimulus at the retina and RPE/Choroid [28, 29]. We observed that the phenotype of peripheral myeloid infiltrating cells varies with time and depth in the detached retina. Uninjured retinas showed elongated EGFP^+^ cells which most likely carry patrolling functions. At 24 hours following RD, early retinal infiltrating cells have an ameboid round morphology, most likely due to the early transmigration from retinal capillaries [30]. This morphology can be observed consistently at the superficial, deep, and subretinal layers. On day 7 following RD, as monocytes differentiate into tissue-resident macrophages, they acquire a ramified phenotype in the superficial and deep retinal layers, which may indicate a modulatory or resting state, as previously described [31]. However, myeloid-derived subretinal cells were substantially larger and adopted a round ameboid morphology, most likely given their phagocytic function [31]. Similar morphological changes have been described by Kaneko et al. [15]. In conclusion, peripheral cells acquire different morphology and possibly different functions, at different retinal layers and RD phases.

Regarding, this peripheral immune cell infiltration, as the retina is detached, myeloid cells infiltrate the retina and subretinal space. Kaneko et al. have shown in retinal cryosections that transplanted peripheral bone marrow cells infiltrated exclusively around the detached retina 4 weeks following detachment [15]. However, we found that the attached retina showed some marginal peripheral infiltration on day 3 (Fig. 2B). It is challenging to grasp the potential outcomes of this early infiltration of the attached retina. Of note, studies have suggested visual decline in macula-on RD [32, 33]. Di Lauro et al. showed in a prospective multicentric study that about 14.9% of macula-on RD patients experienced visual decline after successful reattachment surgery [33]. Despite the pre- and intra-operative variables that may contribute to this effect given the above-mentioned deleterious effects of peripheral immune infiltration on photoreceptor survival, we speculate that this marginal infiltration may contribute to photoreceptor dysfunction and subsequent visual impairment in the attached retina.

Using flow cytometry analysis in the detached retina, Kiang and colleagues found that microglia and myeloid cell numbers were significantly increased at 3 days after detachment, while not at day 1 [34]. However, we cannot conclude that the number of retinal microglia changes with RD, since no microglia-specific marker or proliferation assays were presented. In a similar experimental model, Conart et al. described a rapid infiltration of CD11b^+^ CD45^hi^ Ly6G^+^ and CD11b^+^ CD45^hi^ Ly6G^−^ Ly6C^hi^ cells, peaking at day 1 in the detached retina [35]. We observe a similar pattern in Ly6G and Ly6C^+^ cells, however, we observe a sustained infiltration of the detached retina with a significantly higher CD11b^+^ population at day 7, in flow cytometry samples and retinal wholemounts.

The cellular and molecular events triggered in retinal detachment have been compared to a wound-healing response [36–38]. The cytokine cascade, leukocyte migration, and extracellular matrix remodeling are features of both processes [38]. The migration of peripheral leukocytes upon injury is characterized by early infiltration of neutrophils and consecutive monocyte migration. The neutrophil and monocyte/macrophage infiltration evidenced in this injury model parallels this inflammatory cellular recruitment paradigm. We observed that subretinal neutrophils peak on day 1, whereas monocytes/macrophages on day 3 after retinal detachment. It is interesting to note that no other cell was found in the subretinal space, which suggests a predominant role of the innate immune response in this model.

Systemic monocyte/macrophage depletion with clodronate liposomes significantly reduced infiltrating CD11b^+^ cells on days 1 and 7. Using this approach, Drabek et al. have demonstrated that clodronate liposomes do not cross the blood–brain barrier [26]. Moreover, Peng et al. have shown that this approach does not deplete microglia in the retina [24] or central nervous system [25]. Taken together, this suggests that the observed photoreceptor protection seen in our work corresponds to peripheral and not spurious microglial depletion. Interestingly, monocyte/macrophage depletion has been shown to be protective of the outer retina in sodium iodate (NaIO_3_)-induced retinal degeneration [39]. However, this model induces a combined outer retina and RPE degeneration. Therefore, it is cumbersome to dissect the effect of monocyte/macrophage depletion amid the concomitant degeneration of the outer retina and RPE with NaIO_3_. In our work, the induced experimental retinal detachment causes photoreceptor degeneration exclusively, without associated RPE cell death. Altogether, this indicates that peripheral monocyte/macrophage depletion is protective to the photoreceptors regardless of the RPE status.

The effects of neutrophil depletion have been investigated in other models. Kurimoto et al. have shown that neutrophils can promote axon regeneration in an optic crush injury model, and their depletion suppresses this process [40]. In addition, neutrophil depletion has been studied in wound healing. Dovi et al. have shown that epidermal wound healing was significantly faster in neutrophil-depleted mice compared to controls [41]. In our work, we found that despite the theoretical advantage of neutrophil-mediated debris clearance, depletion of these cells showed increased photoreceptor survival. We found that besides Ly6G+ cell depletion 24 hours following RD, CD11b+ cells showed a similar reduction. It is important to note that neutrophil depletion was performed with the anti-Ly6G antibody (clone 1A8), which specifically depletes Ly6G^+^ cells, without cross-reactivity with Ly6C^+^ [42]. In the bone marrow, monocytes are transiently Ly6G^+^, while mature monocytes express Ly6C but not Ly6G [43]. Altogether, this suggests that the anti-Ly6G (1A8) antibody depletes mature neutrophils and not monocytes/macrophages. Therefore, we speculate that the protective effect of neutrophil depletion may promote a parallel decrease in CD11b^+^ cell infiltration.

This work has several limitations. We used a pharmacological approach to deplete monocytes and neutrophils. We chose this alternative given the lack of commercially available conditional murine models for these populations. In addition, flow cytometry analysis showed lower fold changes compared to wholemount analyses. This may be caused by the required sample pooling, processing, or technical shortcomings of flow cytometry, which may also conceal biological replicate variation.

In summary, the present work demonstrates that the peripheral immune system plays an important role in photoreceptor degeneration after retinal detachment. Monocyte/macrophage and neutrophil depletion are protective for photoreceptor survival. These findings illustrate the potential role of systemic therapeutics to further abrogate visual loss following retinal detachment. Further studies are needed to delineate the contribution of monocytes/macrophages and neutrophils in retinal detachment. Modulation of the innate immune system will likely promote photoreceptor neuroprotection.

### Reporting summary

Further information on research design is available in the Nature Research Reporting Summary linked to this article.

### Supplementary information

Supplementary Table 1

Reporting summary

## Data Availability

The data that support the findings of this study will be openly available in the Open Science Framework from the Center for Open Science at https://osf.io/umvf9/.
