# Supplementary material for: Peripheral monocytes and neutrophils promote photoreceptor cell death in an experimental retinal detachment model
Source: Cell Death Dis. 2023 Dec 16;14(12):834. doi: 10.1038/s41419-023-06350-6 (PMC10724298; doi:10.1038/s41419-023-06350-6)
Supplement: Supplementary file 1 — Supplementary Table 1 [file 41419_2023_6350_MOESM1_ESM.docx]

**Supplementary Table 1.** Reagents, manufacturers, and corresponding catalog numbers.

| **Animal Strain** | **Manufacturer** | **Catalog #** |
| --- | --- | --- |
| C57BL/6J | The Jackson Laboratory | 000664 |
| CCR2^RFP/+^ CX3CR1^GFP/+^ | The Jackson Laboratory | 032127 |
| C57BL/6-Tg(CAG-EGFP)131Osb/LeySopJ | The Jackson Laboratory | 006567 |

| **Reagent** | **Manufacturer** | **Catalog #** |
| --- | --- | --- |
| Provisc | Alcon | ALC-8065183085 |
| 2,2,2-tribromoethanol | Sigma-Aldrich | 152463 |
| 2- methyl-2-butanol | Sigma-Aldrich | T48402 |
| Proparacaine hydrochloride 0.5% | Akorn | NDC 17478-263-12 |
| Hamilton Syringe 701 RN SYR | Hamilton | 7635-01 |
| Bacitracin ointment | Perrigo | NDC 0574-4022-35 |
| Tissue-Tek* O.C.T. Compound | Sakura | 25608-930 |
| Busulfan | Cayman | 14843 |
| ApopTag® Fluorescein In Situ Apoptosis Detection Kit | Millipore Sigma | S7110 |
| Papain Dissociation System | Worthington | LK003150 |
| Tissue-Tek* O.C.T. Compound | Sakura | 25608-930 |
| DAPI | Roche | 10236276001 |
| CD11b clone M1/70 | BioLegend | 101243 |
| CD45 clone 30-F11 | BioLegend | 564279 |
| Ly6C clone HK1.4 | BioLegend | 755194 |
| Zombie NIR | BioLegend | 423105 |
| Clodronate liposomes | Liposoma | C-005 |
| InVivoPlus anti-mouse Ly6G | BioxCell | BP0075-1 |
| RBC Lysis Buffer | Biolegend | 420301 |
| EDTA | ThermoFisher | AM9261 |
| Sodium Azide | Sigma-Aldrich | 71289 |
